# Detection of Four Distinct Volatile Indicators of Colorectal Cancer using Functionalized Titania Nanotubular Arrays

**DOI:** 10.3390/s17081795

**Published:** 2017-08-04

**Authors:** Dhiman Bhattacharyya, Pankaj Kumar, Swomitra K. Mohanty, York R. Smith, Mano Misra

**Affiliations:** 1 Department of Metallurgical Engineering, University of Utah, Salt Lake City, UT 84112, USA; dhiman.opel@gmail.com (D.B.); pankaj.kumar@utah.edu (P.K.); swomitra.mohanty@utah.edu (S.K.M.); york.smith@utah.edu (Y.R.S.); 2 Department of Chemical Engineering, University of Utah, Salt Lake City, UT 84112, USA

**Keywords:** sensor, colorectal cancer, volatile organic biomarkers, titania nanotubes, cancer biomarkers

## Abstract

Screening of colorectal cancer is crucial for early stage diagnosis and treatment. Detection of volatile organic compounds (VOCs) of the metabolome present in exhaled breath is a promising approach to screen colorectal cancer (CRC). Various forms of volatile organic compounds (VOCs) that show the definitive signature for the different diseases including cancers are present in exhale breathe. Among all the reported CRC VOCs, cyclohexane, methylcyclohexane, 1,3-dimethyl- benzene and decanal are identified as the prominent ones that can be used as the signature for CRC screening. In the present investigation, detection of the four prominent VOCs related to CRC is explored using functionalized titania nanotubular arrays (TNAs)-based sensor. These signature biomarkers are shown to be detected using nickel-functionalized TNA as an electrochemical sensor. The sensing mechanism is based on the electrochemical interaction of nickel-functionalized nanotubes with signature biomarkers. A detailed mechanism of the sensor response is also presented.

## 1. Introduction

In the United States, colorectal cancer (CRC) is the fourth most common type of cancer. Despite rapid growth in its diagnosis and therapy, colorectal cancer is still a major burden for cancer mortality. Studies [1,2] indicate that prognosis of the patient can improve considerably if the cancer is recognized at an early stage. This is only possible through a widespread screening for CRC. Early diagnosis and therefore, early treatment and preventive measure is crucial in improving the CRC mortality rate.

In the most cases, the evaluation of patients for CRC uses tissue analysis achieved through complicated and expensive biopsy procedures [3]. Recently, the use of volatile organic compounds (VOCs) with relatively high vapor pressure to detect cancer, emerged as an attractive technique due to its simple and cost effective procedure [3,4,5,6,7,8]. VOCs are present in body fluids such as blood, serum, urine, sweat, and breathe, which can be easily collected and analyzed. This approach is based on the hypothesis that pathological process occurs as a consequence of cancer cells, produces new or changed concentrations of VOCs that are produced normally [9]. It is reported that during the initial stage of cancer (tumor growth), the cancer cell creates the acidic environment which allows the basement membrane to break and allow the access of VOCs to the blood stream [10]. The acidic environment at the same time protects the cancer cell from the immune system to grow tumors. The tumor growth is accompanied by gene changes leading to specific VOCs secreted in the blood fluid that can be utilized to detect specific cancer [11,12]. Studies have demonstrated the production of unique VOCs according to the cancer type due to specific human leukocyte antigen (HLA) forms associated with the different types of cancer [13,14]. It is hypothesized that VOCs are first released from the cancer cells into the bloodstream, then reach the alveoli and are finally exhaled with breath [9,14].

A noteworthy feature of VOCs is the vast array of samples from where they can be extracted. VOCs pertaining to CRC can be extracted from blood, urine, stool, and breath of patients, with each sample type showing certain unique identifying biomarkers. Several studies have investigated the VOCs present in various body fluids associated with CRC. Wang et al. [15] identified four VOCs present in blood samples by examining the blood from 16 CRC patients and 20 healthy subjects using GC-MS. Several other groups have worked to identify VOCs present in urine samples [16]. Silva et al. [17] identified fifteen VOCs in urine samples related to CRC patients. Arasaradnam et al. [8] highlighted the importance of VOCs in the urine and the potential they hold for future noninvasive techniques in this area.

Although urine- and blood-based VOCs have been studied in some details, breath-based VOCs have not been investigated highly. Although, the breath analysis technique to identify cancer is still in an infant stage, recent work has shown the potential to use VOCs in the breath as a signature for cancer identification. In this respect, various VOCs have been detected in the breath for the identification of diverse types of cancer including esophageal cancer, lung cancer, breast cancer, prostate cancer and colorectal cancer. Altomare et al. [4] identified fifteen VOCs as potential biomarkers existing within the breath of colorectal cancer patients using GC-MS techniques that can be used to potentially screen the CRC with 80% accuracy. Six VOCs related to CRC in breath were identified and reported by Peng et al. [3]. Wang et al. [18] and Amal et al. [19] reported nine and four VOCs in exhaled breath to identify CRC, respectively. Among all the VOCs identified, four VOCs, viz. cyclohexane, methylcyclohexane, 1,3-dimethylbenzene and decanal demonstrated a recognition capability. In the present study, Ni-deposited TiO_2_ nanotubes were used to detect the presence of cyclohexane, methylcyclohexane, 1,3-dimethylbenzene and decanal vapors under ambient conditions.

## 2. Background of Noninvasive Screening System for CRC

In a study by Peng et al. [3], they investigated the ability of a cross-reactive nanosensor array based on organically functionalized gold nanoparticles (GNPs) to discriminate between breath VOCs of healthy controls and the patients suffering from lung, breast, colorectal, and prostate cancers. Their arrays of broadly cross-reactive GNP sensors were ideally suited to trace the cancer odor prints directly, without identifying the constituent VOCs, but in reality, the different cancer types mentioned have distinct odor prints comprising succinctly distinct VOCs. Hence, it is evident that the GNP-based sensor suffers from selectivity and specificity issues. From a point-of-care diagnostic standpoint, specificity, selectivity, and sensitivity are critical attributes of any sensing platform. Developing a VOC-based sensing platform for rapid detection of any cancer type disease can significantly impact screening of patients at an early stage, which can otherwise prove to be life threatening. Given that colorectal cancer (CRC) is the second leading cause of cancer-related death in Europe and the third in the USA, the quest for novel noninvasive screening systems with the potential for high patient compliance and low cost that have an equivalent sensitivity/specificity to colonoscopy, for early detection of colorectal cancer and precursor adenomatous polyps continues [6,20,21].

The capabilities of electrochemically-based vapor sensors in this type of setting are highly contingent on the effect of functionalization of an inert but stable substrate base. Metal oxides that are used in gas sensing purposes allow for the surface absorption and desorption of certain gases facilitated by electron transfers on the film surfaces. Studies have demonstrated the application of metal oxides in the detection of VOCs present in gas phase related to CRC [22,23]. In a recent study, VOCs relevant to CRC such as 1-iodononane, benzene and decanal were shown to be detectable in stool samples using various semiconducting metal oxide films [22]. The mixed oxide of titanium (Ti), tantalum (Ta), and vanadium (V) film demonstrated to be better at detection of benzene, while tin and titanium mixed oxide was observed to have good detection capability for 1-iodononane and decanal. While these materials have demonstrated the detection of VOCs relevant to CRC, they require high temperatures (up to 650 °C) for operation, which requires more power. In addition, there are no true specific “recognition elements” for absolute selectivity. For example, in the same study, the mixed oxides of Ti, Ta, and V were shown to be effective for methane gas detection as well. Recently, the polymer based electronic nose (e-nose) non-invasive sensor technology has shown to be useful in screening CRC patients by VOCs detection in fecal gas [24]. Alternatively, an increase in gas sensitivities and selectivity of the metal oxides can be achieved by the metal deposition on oxide substrates [25,26]. It has been reported by several groups that the adjustment of TiO_2_ with certain metallic depositions can alter the band gap of it [27,28,29], allowing for greater conductivity patterns. To that effect, it has been reported by Bhattacharyya et al. [30] that TiO_2_ in a crystalline anatase phase confers greater mechanical strength and stable base for such catalytic processes. It was further reported that the utility of TiO_2_ based gas sensors lies in their applicability in high and low temperature settings due to the versatility in the morphology of the surface of the TNA [31]. With a high surface area-to-volume ratio [30,32], corrosion resistance [33], and unique mechanical characteristic, it is possible to increase the susceptibility of these sensors by functionalization.

Previous studies have demonstrated the feasibility of utilizing cobalt functionalized titania nanotube-based detection of tuberculosis biomarkers found in breath [30,32,34]. Prior to examining CRC biomarkers in breath using TiO_2_ nanotubes, it is imperative to determine the functionalization element, which can facilitate catalytic oxidation/reduction of the biomarkers. The catalytic oxidation of cyclohexane and similar hydrocarbons has been of great interest for its application in the industrial sectors. Adipic acid, a product of such oxidative processes, is a vital component in the synthesis of Nylon-6 and Nylon-66 [35]. Several techniques to achieve this have been developed as a result. For instance, Alshammari et al. [35] reported on their success in a direct catalytic conversion of cyclohexane to adipic acid with nanogold catalysts, while Sarkar et al. [36] reported on a room temperature conversion using copper nanoparticles deposited on a Cr_2_O_3_ substrate. Hybridized substrate bases of WO_3_/V_2_O_5_ with a generic oxidant H_2_O_2_ have also been used in this catalytic system [37]. Photocatalytic conversion of cyclohexane to adipic acid has also been reported as a more green method of synthesis with the utilization of TiO_2_ functionalized with iron, nickel, and gold [38]. Gaur et al. [39] demonstrated the catalytic oxidation of 1,3-dimethylbenzene using activated carbon fiber impregnated with nickel. In addition, Yolcular et al. [40] show that Ni/Al_2_O_3_ catalysts exhibits activity in the dehydrogenation of methylcyclohexane for hydrogen production. Using this study as well as the reviewed publication by Hosseini et al. [33], nickel was determined to be a highly active oxidant species capable of the catalytic conversion of biomarkers of interest. This evidence is further supported by the study of Li et al. [41] that explored the potential of nickel-deposited TiO_2_ in a variety of hydrogen sensing purposes. In their study, Ni-TiO_2_ catalyst demonstrated good sensing capabilities in hydrogen environments of varying concentration over a wide temperature range (25 °C–200 °C).

## 3. Materials and Methods

The procedure for the synthesis of titania nanotubes has been explained in detail in our previously published work [30,32,42]. Annealed TiO_2_ nanotubes were electroplated with a 0.5 M NiCl_2_ solution at room temperature. The cathodic deposition of Ni ions was carried out at 22.5 mA, with a current density of 10 mA/cm^2^ for 1 min. A Pt foil was used as the anodic counter electrode; the two electrodes were held approximately 2.5 cm apart. The Ni-deposited TiO_2_ nanotubes were then rinsed with DI water and left to dry overnight in an oven (VWR, Radnor, PA, USA) at 110 °C under vacuum.

The morphological characteristics of the TNA and Ni-deposited TNA were analyzed with a S-4800 SEM (Hitachi, Chiyoda, Tokyo, Japan) consisting of a tungsten film of accelerating voltage of 5.0 kV and an emission current setting of 15 µA. In addition to the SEM, an attached Oxford EDX detector (Abingdon, UK) provided energy dispersive spectroscopic (EDS) analysis of the corresponding SEM images. These were done at 20 kV accelerating voltage on a 25 × 25 µm area.

XPS studies were carried out using an Axis Ultra DLD model instrument (Kratos Analytical, Wharfside, Manchester, UK). The vacuum inside the analysis chamber was held at a constant 3 × 10^−8^ Torr. A monochromatic AlK_α_ radiation at 180 W and h*v* = 1486.6 eV was used as the x-ray excitation source. The spectra were obtained at passing energies of 160 and 40 eV, and were analyzed with the aid of CasaXPS software. The C 1s line at 284.6 eV was used as the standard reference for all the peaks.

X-ray diffraction (XRD) was carried out using a Miniflex XRD (CuKa = 1.54059 Å, Rigaku, Akishima-shi, Tokyo, Japan) from 2Theta = 20 to 90 degrees with a step size of 0.015 degrees and dwell time of 1 degree min^−1^. The diffraction patterns were analyzed using Rigaku PDXL2 analysis software and indexed with standard JCPDS cards.

The electrocatalytic oxidation of the four CRC biomarkers for their detection was carried out in a customized sensing chamber such that one connection (the working electrode) was in contact with the Ni-deposited TNA side, while the other electrode (the counter electrode) was in contact with the unanodized (polished Ti surface) face of the coupon. Contact was achieved using copper clips. A Reference 600 two-electrode based potentiostat (GAMRY, Warminster, PA, USA) was used during these experiments. To introduce biomarker vapor into the sensing chamber, nitrogen gas at 200 standard cubic centimeters per minute (sccm) was bubbled through the solution of the biomarker mimics. A schematic diagram of overall experimental set up for CRC biomarker detection was shown in our previous work [30]. Varying concentrations of biomarkers (0.1, 1, and 10 mM, all dissolved in ethanol) were used in these experiments. From preliminary cyclic voltammetry studies, the bias voltages that allowed for maximal interaction between nickel and the individual VOBs were determined. At the respective voltages, sensor response tests were carried out. During the tests, for the first 100 s, only nitrogen gas (no biomarker) was purged to allow for a minimum current value to be established, followed by exposure to biomarker vapors. When maximum current with the analyte was observed, the biomarker was disconnected and the sensor was exposed to only to nitrogen gas again where a drop in the current was visible. All the experiments were conducted at room temperature.

## 4. Results and Discussion

### 4.1. TNA Surface Characterization

**Figure 1:**
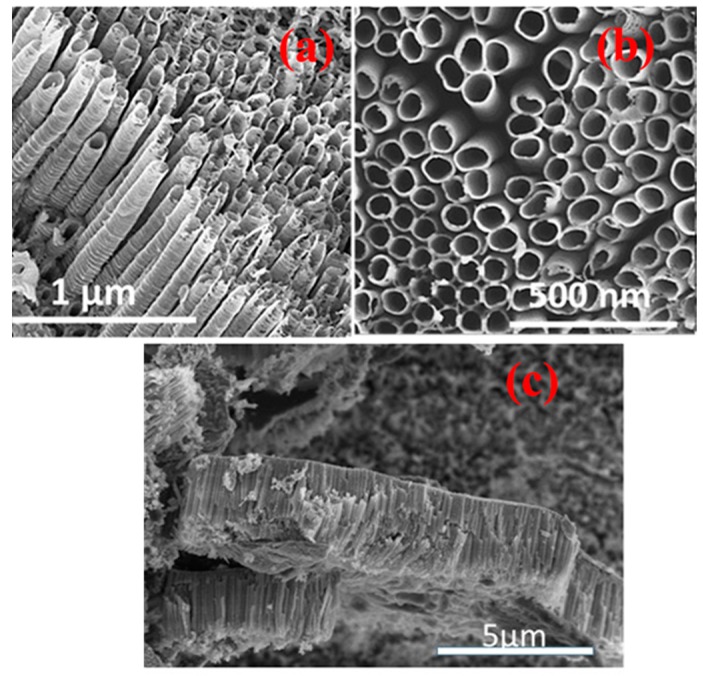
SEM micrographs of highly organized, vertically oriented TiO_2_ nanotubular array: (**a**) side wall view; (**b**) top view; and (**c**) an island chunk of TNA lying on the mouth of the nanotubes.

**Figure 2:**
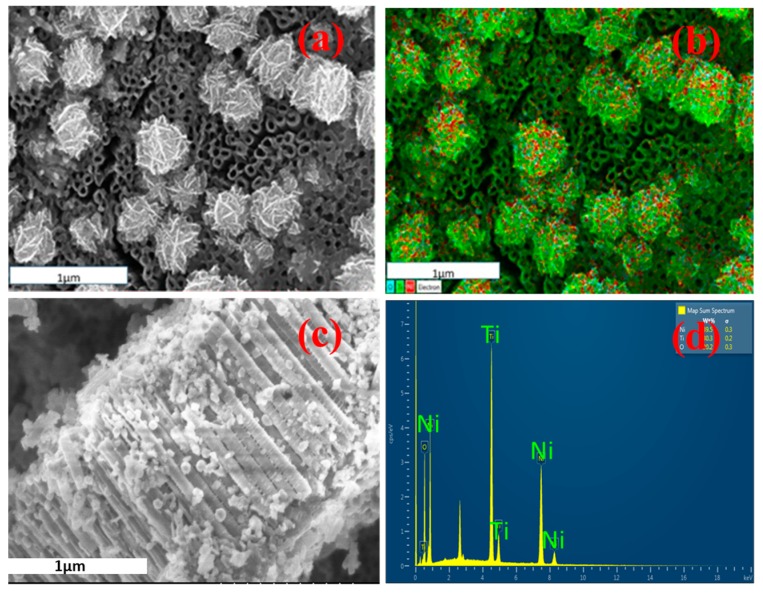
SEM image of (**a**) electrodeposited Ni on TNA; (**b**) EDS micrograph confirming the presence of Ni globules on TNA; (**c**) Ni globules present on the side wall of the nanotubes, (**d**) EDS spectrum further confirming the presence of Ni.

Scanning electron micrographs show that the TNA (Figure 1) is organized into regularly sized and ordered nanotubes. A top side view indicates the tubes having a diameter of approximately 55–60 nm and a wall thickness of approximately 14 nm. From side views of the nanotube array, it is estimated that the length of the nanotubes ranges from 1.2–1.5 µm. The TNA film showed the presence of primarily anatase phase titania, which in combination with annealing in an oxygen environment, showed greater electrical resistivity which is important for electrochemical detection of VOCs. Scanning electron micrographs of Ni-deposited TNA (Figure 2a–c) showed deposition of nickel as globules on the TNA surface.

The globules range anywhere from 0.4–0.6 µm in diameter and can be seen to be relatively homogeneously distributed. Lateral cross-sections reveal that smaller, individual particulates of nickel fit within the openings of the tubes. It is assumed that electroplating of nickel favors congregation of the particulates into globules rather than individual particles on the surface. This confirmation is well suited for sensory purposes due to the higher surface area that is now available for interaction with biomarkers. The EDS spectrum (Figure 2d) shows the presence of titanium, oxygen, and nickel on the TNA surface. EDS mapping of the SEM images (Figure 2b) reveals that the globules consist of titanium, nickel, and oxygen with their approximate weight distributions as 47.0%, 26.8%, and 26.2%, respectively.

**Figure 3:**
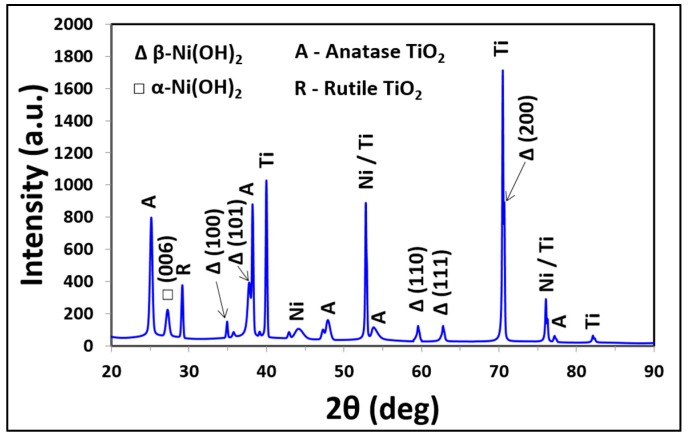
XRD pattern of Ni-TNA with 2θ ranging from 20° to 90°.

The presence of nickel is further supported by the XRD diffraction studies. The XRD diffraction patterns of the Ni functionalized TNA sensor is shown in Figure 3. The peaks in the diffraction pattern correspond to phase of anatase TiO_2_, Ni and Ni(OH)_2_. The pattern, therefore, confirms that the annealed TNA is predominantly crystalline anatase phase. In addition, the peaks of Ni(OH)_2_ are observed indicating that the Ni present as Ni(OH)_2_. Although the diffraction pattern shows the presence of both α- and β-Ni(OH)_2_ phases, the stronger peaks obtained for β-Ni(OH)_2_ indicates to be mainly β-phase. This is attributed to intense (200) and (101) diffraction along with the visible (100), (110) and (111) peaks of β-Ni(OH)_2_. The overlapped Ni and Ti peaks observed in the diffraction pattern at 53° and 77° indicate their distinct existence, and Ni is not integrated within the lattice of the TiO_2_ nanotube array. This is of great importance in regards to the reaction between Ni(OH)_2_ and the biomarkers.

**Figure 4:**
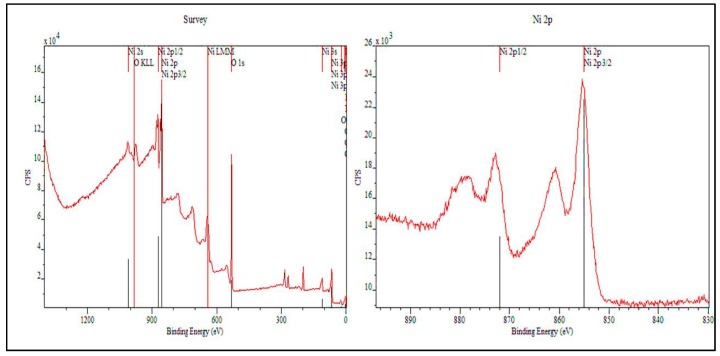
XPS spectra of Ni-TNA (**Left**) General survey of the sample shows numerous peaks with prominent ones for nickel and oxygen. Oxygen’s 1s peak is observable at 532 eV; (**Right**) XPS spectra of Ni shows two prominent peaks of interest: 2p_1/2_ at 872 eV and 2p_3/2_ at 855 eV.

The presence of Ni as Ni(OH)_2_ is further supported by XPS studies (Figure 4). It revealed that the existence of Ni as Ni(OH)_2_ globules on the surface as seen by SEM. A general survey of the sample shows numerous peaks with prominent ones for nickel and oxygen.

Oxygen’s 1s peak is observable at 532 eV. In the case of nickel, there are two prominent peaks of interest: 2p_1/2_ at 872 eV and 2p_3/2_ at 855 eV. These values well are established, for example, Wu et al. [43] concluded that these energy values correspond to Ni(OH)_2_. There is a gap of 17 eV between the peaks of interest, which indicates that the Ni 2p peak has significant split spin-orbit components. A very small gap exists between the 2p and 2p_3/2_ peaks, but due to spin coupling and overlay of similar energy orbitals on the nickel atom, they appear to be as one peak. Near the lower end of the spectrum, 3s and 3p peaks (0–100 eV) are also visible, but due to their low energy values and minor peak sizes, these are assumed to be not participating during the progression of the reaction.

### 4.2. Detection of CRC Biomarkers with Ni-TNA Sensor

The ability of nickel to act as a strong binding agent to the biomarkers was determined from cyclic voltammetry studies. From the same studies, the optimal voltages for the reaction between nickel and the individual biomarkers cyclohexane, 1,3-dimethylbenzene, methylcyclohexane, and decanal were 1 V, 1.4 V, 1.55 V and 1.45 V, respectively. These bias voltages have been observed to produce the maximum current signal when exposed to the respective biomarkers.

**Figure 5:**
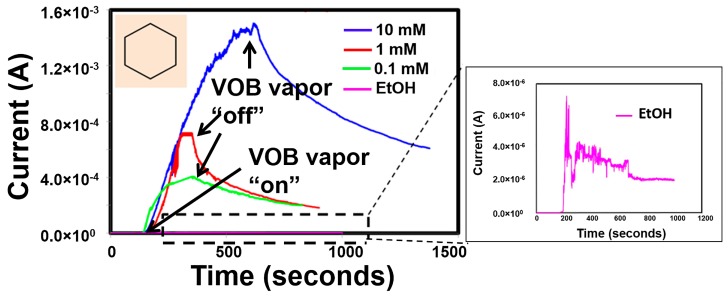
Sensor current response of Ni-TNA to vapors of cyclohexane in solution of varying concentration in ethanol, inset shows the sensor response to ethanol.

Figure 5 shows the current response of Ni-TNA sensor when exposed to the cyclohexane using a bias voltage of 1 V. It can be observed that when the sensor is exposed to the individual biomarkers, there is a sharp and rather rapid increase in current. For a 10 mM concentration of cyclohexane, the peak current output was recorded as 1.45 mA.

As the concentration was decreased by factors of 10, a corresponding decrease in the maximum current recorded was noted; vapors from 1 mM solution gave a response of ~700 µA while a current of 392 µA was obtained from 0.1 mM solution. The estimated sensor response times were ~450 s, ~180 s, ~200 s for 10 mM, 1 mM and 0.1 mM cyclohexane respectively. The response time is defined as the time taken to reach to the maximum sensor current. Since ethanol was the solvent in which this biomarker was dissolved, the reaction binding affinity of nickel to ethanol was also measured as the sensor control response. Compared to cyclohexane, the maximum current observed for ethanol was <3 µA, a significantly lower value (Figure 5 inset). This is indicative of the fact that the large current peaks observed during the experiments were due to exposure of the sensor to cyclohexane and a successive drop in current was observed when the flow of cyclohexane was discontinued. However, the observed current does not recover to its initial current during the course of the experiment. Therefore, the sensor recovery time of the sensor is unknown. The recovery time of the sensor is defined as the time required for the sensor to recover to initial current value when the sensor exposure to the VOB is stopped.

When the sensor is exposed to cyclohexane, the Ni(OH)_2_ on TNA chemically interacts with the cyclohexane to form complex compounds and generate electrons through the redox reaction. The complex formation mechanism is discussed in detail in Section 4.3. It is possible that once the complex compound formed, the surface does not recover to the initial composition during the course of the experiment. The sensor saturates during the VOCs interaction, thus sensor recovery is restricted. However, further study is required to understand sensor recovery phenomena.

**Figure 6:**
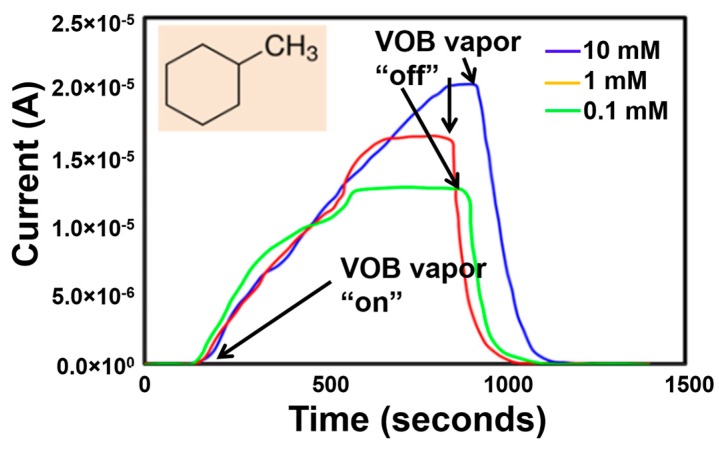
Sensor current response of Ni-TNA to vapors of methylcyclohexane at varying concertation in ethanol solution, arrows show the point when the sensor exposure to the VOB was started and removed.

Similar to cyclohexane, a current response was observed when Ni-TNA sensor was exposed to another CRC biomarker, methylcyclohexane. A bias voltage of 1.4 V was utilized to obtain the current response. The current responses of Ni-TNA due to methylcyclohexane vapor in various concentration solutions are shown in Figure 6. It can be seen that the current increases rapidly as soon as the biomarker vapor is exposed to the sensor. A maximum current response from of ~21 μA was obtained for 10 mM methylcyclohexane solution. The current response reduced to ~15 μA and ~11 μA when the solution concentration reduced was to 1 mM and 0.1 mM. Interestingly, a complete sensor recovery was observed within ~100 s of shutting off the sensor exposure to the biomarkers at all concentration levels.

**Figure 7:**
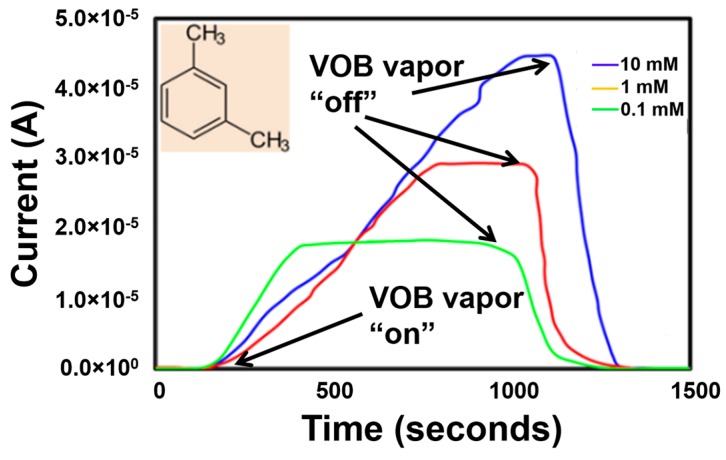
Sensor current response of Ni-TNA to vapors of 1,3-dimethylbenzene at different concertation in ethanol solution, arrows show the point when the sensor exposure to the VOB was started and removed.

A current response was observed for 1,3-dimethylbenzene vapor when exposed to the sensor as well. A maximum current of ~45 μA was obtained from 1,3-dimethylbenzene vapor in 10 mM solution as shown in Figure 7. The current response of the Ni-TNA decreased when the concentration of the solution was reduced. A maximum current of ~28 μA and ~17 μA was obtained from 1 mM and 0.1 mM solutions, respectively (Figure 7).

A complete sensor recovery similar to methylcyclohexane was observed when 1,3-dimethylbenzene exposure to Ni-TNA was stopped. The exact mechanism of sensor recovery due to exposure of specific CRC biomarkers is subject of further study.

**Figure 8:**
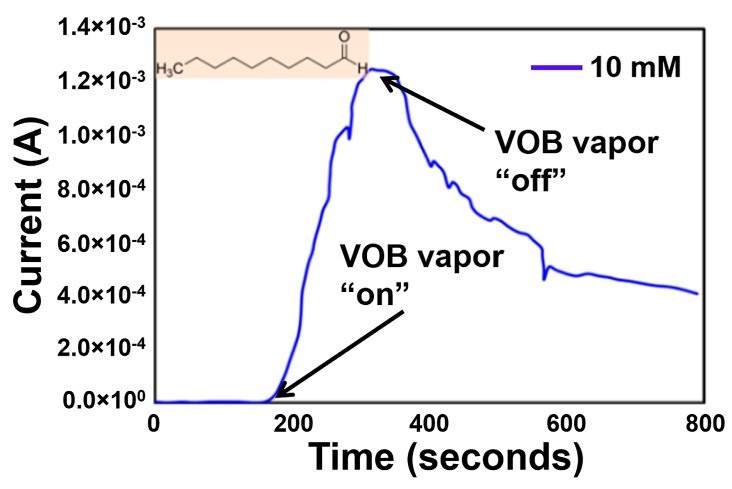
Sensor response of Ni-TNA to vapors of decanal at 10 mM concertation in ethanol, arrows indicate the point when the sensor exposure to the VOB was started and removed.

When the decanal VOB was exposed to Ni-TNA sensor, the current increased rapidly and reached to a maximum current of ~1 mA and reduced when the exposure to the sensor was stopped as shown in Figure 8. This indicates that the decanal vapors from 10 mM solution in ethanol delivered a maximum current of ~1 mA (Figure 8). However, when the biomarker exposure was stopped, the Ni-TNA does not recover to its initial current value. This sensor recovery behavior can be attributed to change in surface chemistry due to biomarker interaction.

The present study shows that Ni-functionalized TNA is sensitive towards all four prominent CRC biomarkers. In addition, the sensor response is directly related to the concentration of the biomarkers in the solution. However, further investigation is required to determine the vapor phase concentration of the biomarkers reaching the sensor substrate.

**Figure 9:**
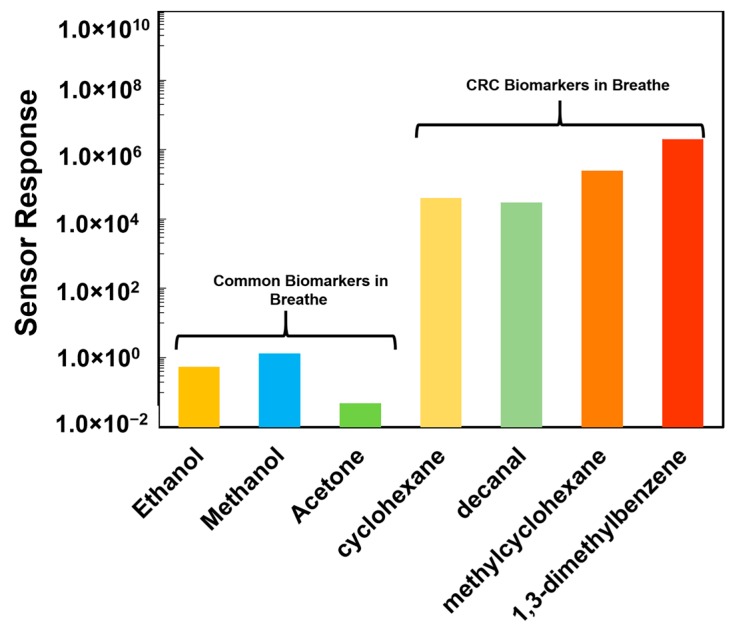
Sensor response of Ni-TNA when exposed to the common VOBs found in breath and signature biomarkers associated with CRC. A very low response was observed when exposed to the more common breath VOBs compared to the sensor response from the CRC biomarkers.

It is well known that the common VOCs such as ethanol, methanol, and acetone are present in the human breath along with the signature CRC biomarkers. Therefore, sensor responses from these common VOCs are imperative to study to understand the sensor selectivity to CRC biomarkers. In the present study, the Ni-TNA sensor response from the common VOCs in breath along with the CRC biomarkers are calculated using the following equation:

(1) $$Sensor\;response\;\left(SR\right)=\frac{i_{max,\;VOC}-i_{max,\;base\;line}}{i_{max,\;base\;line}}$$

where, *i_max,VOC_* is the maximum current when the sensor is exposed to the nitrogen bubbled through VOCs solution and, *i_max,base line_* is the maximum current when exposed to nitrogen gas. To determine sensor response of the common VOCs, the vapors at given concentration (1000 ppm) in water is exposed to Ni-TNA. The obtained sensor response from the ethanol, methanol, and acetone along with signature CRC biomarkers are shown in Figure 9. It can be seen that the sensor response to ethanol, methanol and acetone ranged from 10^−2^ to 10 which is much smaller than the response from the signature biomarkers (10^4^ to 10^6^) operated under similar condition. This results indicative the Ni-TNA sensor to be highly selective towards the CRC biomarkers.

### 4.3. Sensing Mechanism

From SEM and EDS analysis of Ni-deposited TNA, it was found that the TNA surface was littered with globular Ni(OH)_2_ deposits. Since the solution in which electroplating had been conducted was aqueous NiCl_2_, a possible equation for the reaction which explains the formation of nickel hydroxide is following:

NiCl_2_ + 2H_2_O → Ni(OH)_2_ + 2HCl (2)

It has been well reported that the gas sensing capabilities of sensors are based on oxide conductivity adjustments that occur on the surface or near the surface of sensors as a consequence of gas adsorption or the formation of complexes [30,38,44]. In the case of organic species like cyclohexane, the formation of complexes is the primary mechanism for detection. We have previously reported the formation of a complex between Co^2+^ ions and biomarker methyl nicotinate in a similar procedure [30]. In both cases, the presence of metal ions is not only crucial for the stability and formation of complexes but also the ability to act as oxidizing agents in these reaction mechanisms.

From previous discussions of various methods of cyclohexane oxidation, there are two reaction mechanisms by which the conversion of cyclohexane to adipic acid is facilitated. In both of the reactions, whether it be the direct conversion to adipic acid, or the formation of the intermediate product cyclohexanol and cyclohexanone [37], the production of the cyclohexyl radical is of great importance. To that effect, Ide et al. [38] reported in their photocatalytic experiments that the synthesis of this intermediate radical on TiO_2_ is carried out by the direct interaction of cyclohexane with either the valence band hole of TiO_2_ or a combination of the valence band hole and its reduction of an OH^−^ group. It is well known that TiO_2_ is an n-type semiconductor and being as such, the interaction of valence band holes and cyclohexane will be minimal. This is supported from our cyclic voltammetry studies (not shown) where it was found that functionalization of TiO_2_ was required for the detection of cyclohexane since TiO_2_ within itself was an inadequate oxidizing agent in electrochemical settings. However, the formation of Ni(OH)_2_ complexes on the surface of the TNA, more specifically the OH^−^ groups, was crucial in this reaction. The presence of OH^−^ groups on the surface increase the general conductivity of n-type sensors. This fact is supported by our previous studies that have reported not only the utility of OH^−^ as great Brönsted-Lowry acids but also their defining role in the stability of these organometallic complexes. Because of the presence of OH^−^ on the TNA surface, the formation of a stable complex with cyclohexane vapor is possible.

**Figure 10:**
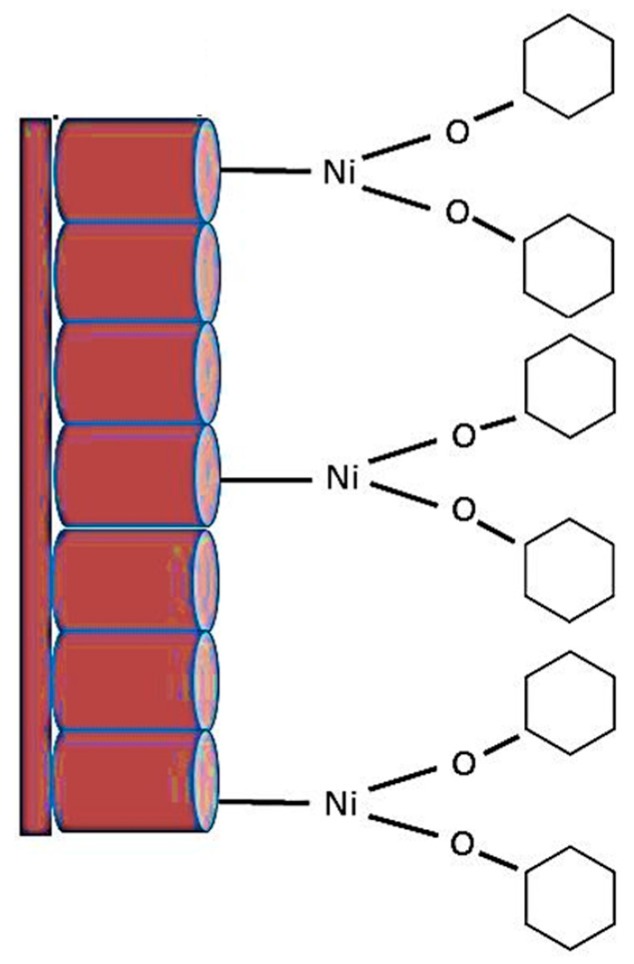
Schematic diagram showing the formation of the nickel cyclohexyl complex on the TNA*.*

The reaction between the Ni(OH)_2_ complexes and cyclohexane can be envisaged in Equation (3). Due to the nickel functionalization of the TNA, cyclohexane gets oxidized and creates a nickel cyclohexyl complex with two cyclohexane molecules attached to one nickel atom (Figure 10).

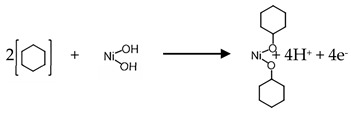
(3)

The high current response, indicative of a strong interaction between nickel and cyclohexane, is well corroborated by the reaction mechanism shown above. Not only does the efficiency of binding follow a 1:2 ratio of nickel to cyclohexane, but the unique characteristics of TiO_2_ nanotubes in electron transfer and atmospheric oxygen adsorption make it a suitable and highly favorable reaction. In the case of the former, as reported by Bhattacharya et al. [30], the structure of these nanotubes provides not only stability and increased surface area of the interaction between the metal ions and cyclohexane but the tubular structure also enables greater electron transfer between the biomarker and the metal ions during the oxidative interaction with minimal losses. As to the adsorption of atmospheric oxygen, this characteristic of TiO_2_ is vital in the steps following the synthesis of this radical complex that allows for the formation of the intermediates mentioned above [37,38]. This is supported by the studies of Ide et al. [38] that reports on the importance of the superoxide anion that plays a role in the formation of cyclohexanone and cyclohexanol. TiO_2_ generates this superoxide anion by donating its electrons to the ambient atmospheric oxygen that is present during the progress of this reaction. The following equations reported by Bhattacharya et al. [30] show how this may be possible:

O_2_ (gas) → O_2_ (ads) → O^2−^ (ads) → 2O^−^ (ads) → O^2−^ (ads) (4)

Oxygen vacancy (Vo)-related defect sites/states are the most favorable ones for the adsorption of target species [45,46]. This is because the binding interaction between oxygen molecules and organic molecules is much stronger in such defect sites compared to the defect-free ones. O_2_ molecules can be dissociated and chemisorbed to the oxygen vacant site (Vo) of the oxide surface with negligible activation energy [45,47]. It is speculated that once the nickel-cyclohexyl complexes have been created, the superoxide anion is responsible for the direct conversion of the cyclohexyl radical to cyclohexanone and cyclohexanol. This is corroborated by the sensor response graphs (Figure 5) that show a remarkably slow decrease in current after the discontinuation of cyclohexane vapor flow. This is indicative of the possibility that less and less cyclohexyl radical is available for reduction back to cyclohexane. The current signal that has been reported during this experiment may be a result of this reaction mechanism.

Similar reaction mechanisms have been envisaged for the interaction of Ni with other biomarkers as well (Equations (5)–(7)). The reaction with 1,3-dimethylbenzene and methylcyclohexane shows that the organic molecules doubly and triply complexes with nickel hydroxide, respectively. As a result, a single organic molecule occupies effectively more active sites and fewer active sites are thus available for other unreacted molecules. The maximum current response to 10 mM of the respective vapors is also in the same order: 45 μA for 1,3 dimethylbenzene, and 21 μA for methylcyclohexane. As the reducing gas/vapor comes to contact the sensing layer, it is adsorbed to the oxide surface by the dissociative adsorption process and dehydrogenated by the adsorbed oxygen species. Decanal is a straight chain molecule and attaches to a single molecule. Therefore, in addition to not providing any steric hindrance, more active sites are available for unreacted species to form nickel complexes. These results in higher current response (~1 mA) to 10 mM vapors of decanal in comparison to the previous two recently discussed. Further, catalytic activity of TiO_2_ enhances the dissociation possibility of the aldehydes (decanal) at the oxide surface. The effect of TiO_2_ catalyst converts aldehyde to a secondary alcohol. In alcohols, O−H bond scission becomes faster, and alcohols can easily be dissociated and dehydrogenated into different alkoxy fragments [31]. These species are adsorbed to the TiO_2_ nanotube surface occupying two active defect sites according to the following reaction (Equation (5)) [45]. Because of the capture of the free electron to the oxide surface, sensor resistance decreases effectively [45].

RCHO(vapor) → ROH(vapor) + O^−^(ads) → [RO]^−^(ads) + OH(ads) (5)

where R represents the alkoxy group. Subsequently, the adsorbed alkoxy fragments recombine with the adsorbed hydroxyl ions and form the molecular alcohols, releasing a free defect site following the reversible process as:

[RO]^−^(ads) + OH^−^(ads) → ROH(vapor) + O(ads) (6)

Two adsorbed hydroxyl groups then react with each other and release water and a free electron (offering a free defect site) following the reaction:

OH^−^(ads) + OH^−^(ads) → H_2_O + O(ads) + e^−^ (7)

The adsorbed alcohol (ROH) molecules can also be directly oxidized or dehydrogenated by the surface-adsorbed oxygen releasing free electrons (offering free defect sites) to the oxide surface that effectively reduces the sensor resistance of the n-type TiO_2_ nanotube arrays [31].

During room temperature sensing, adsorption of the alkoxy groups on the nanotube surface strongly depends on the availability of oxygen vacancy sites and the surface coverage of the alkoxy groups. At any particular biomarker concentration, when surface coverage of the alkoxy groups is almost constant, the response magnitude of a sensor is principally dominated by the availability of the free oxygen vacant sites (Vo). Thus, we observe that the magnitudes of the amperage sensor response of Ni-TNA to the four CRC biomarkers are pertinent to the reaction mechanism proposed for each.

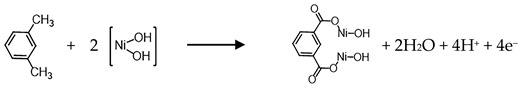
(8)

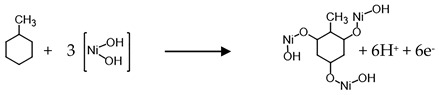
(9)

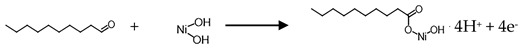
(10)

## 5. Conclusions

Nickel-functionalized TNAs have been demonstrated to be strong candidates in the detection of CRC biomarker vapors. To account for the recorded sensor response, a mechanism has been proposed. The mechanism assumes the role of Ni(OH)_2_ as a catalytic oxidizing agent capable of oxidizing the CRC VOCs with the combined force of the valence band holes of TiO_2_ and the reduction of radical OH^−^ groups. This leads to the formation of a complex between nickel and the cyclohexyl radicals that are then further oxidized by the superoxide anion generated by TiO_2_ by reducing atmospheric O_2_ gas with the conduction valence band. This study establishes the Ni-TNA sensor can be utilized for amperometric detection of signature CRC biomarkers with the aid of nickel as an oxidizing agent and provides a platform for future exploration in the field of VOCs and the implications of VOC detection in CRC diagnostics.
